# Anifrolumab in lupus nephritis: results from second-year extension of a randomised phase II trial

**DOI:** 10.1136/lupus-2023-000910

**Published:** 2023-08-22

**Authors:** David Jayne, Brad Rovin, Eduardo Mysler, Richard Furie, Frédéric Houssiau, Teodora Trasieva, Jacob Knagenhjelm, Erik Schwetje, Weifeng Tang, Raj Tummala, Catharina Lindholm

**Affiliations:** 1 Department of Medicine, University of Cambridge, Cambridge, UK; 2 Department of Internal Medicine–Nephrology, The Ohio State University, Columbus, Ohio, USA; 3 Rheumatology, Organizacion Medica de Investigacion SA, Buenos Aires, Argentina; 4 Division of Rheumatology, Donald and Barbara Zucker School of Medicine at Hofstra/Northwell, Great Neck, New York, USA; 5 Rheumatology Department, Cliniques universitaires Saint-Luc, Institut de Recherche Expérimentale et Clinique, Universite catholique de Louvain, Brussels, Belgium; 6 BioPharmaceuticals R&D, AstraZeneca, Goteborg, Sweden; 7 BioPharmaceuticals R&D, AstraZeneca, Gaithersburg, Maryland, USA

**Keywords:** autoimmune diseases, lupus nephritis, therapeutics

## Abstract

**Objective:**

To characterise the safety and efficacy of anifrolumab in active lupus nephritis (LN) through year 2 of the phase II randomised, double-blind Treatment of Uncontrolled Lupus via the Interferon Pathway (TULIP)-LN trial (NCT02547922) of 2 anifrolumab dosing regimens versus placebo.

**Methods:**

Patients received intravenous anifrolumab 900 mg for the first 3 doses followed by 300 mg anifrolumab (intensified regimen (IR)), 300 mg anifrolumab (basic regimen (BR)) or placebo every 4 weeks throughout. To continue into Year 2, patients must have achieved at least partial renal response and a glucocorticoid tapering target.

**Results:**

Of 147 randomised patients, 101 completed Year 1 study treatment; of these, 75 (74%) continued into Year 2 (anifrolumab IR: n=29, BR: n=23 and placebo: n=23). During Year 2, 72% of patients reported ≥1 adverse event (AE); serious AEs were reported in 6.9%, 8.7% and 8.7% of patients (anifrolumab IR, BR and placebo, respectively); 3 patients discontinued treatment due to an AE (anifrolumab IR: n=2 and placebo: n=1) and herpes zoster was reported in 2 patients (anifrolumab IR: n=1 and BR: n=1). The study was ongoing at the start of the pandemic, but no COVID-19 cases were reported. Of the 145 patients receiving treatment, more patients on the IR attained complete renal response at Week 104 compared with those on BR or placebo (27.3% vs 18.6% and 17.8%) and simultaneously achieved sustained glucocorticoid tapering (IR: 25.0%; BR: 18.6% and placebo: 17.8%). The improvements in estimated glomerular filtration rate were numerically larger in both anifrolumab groups versus placebo.

**Conclusions:**

The safety and tolerability profile through Year 2 of TULIP-LN was generally consistent with Year 1, with promising efficacy results for the anifrolumab IR regimen. Collectively, the results support further investigation of an anifrolumab intensified dosing regimen in larger populations of patients with active proliferative LN.

**Trial registration number:**

NCT02547922.

**WHAT IS ALREADY KNOWN ON THIS TOPIC**

- Anifrolumab targets the type I interferon (IFN) signalling pathway by specifically blocking the type I IFN receptor, which is known to be involved in lupus nephritis (LN) pathogenesis.
- The primary outcome of the first year of the phase II TULIP-LN randomised, placebo-controlled trial suggested that an intensified regimen (IR) of anifrolumab has potential to be a novel treatment option for patients with active LN.

**WHAT THIS STUDY ADDS**

- This 2-year analysis of the placebo-controlled TULIP-LN study shows acceptable long-term safety and tolerability of anifrolumab.
- Treatment with anifrolumab using an IR dosing regimen added to standard of care with mycophenolate mofetil and glucocorticoids, improved renal and non-renal disease outcomes in patients with active class III or IV LN.

**HOW THIS STUDY MIGHT AFFECT RESEARCH, PRACTICE OR POLICY**

- These results support investigation of the anifrolumab IR dosing regimen in patients with active class III or IV LN in the ongoing phase III IRIS study (NCT05138133).

## Introduction

Lupus nephritis (LN) is among the most common severe organ manifestations of systemic lupus erythematosus (SLE), occurring in up to ∼50% of patients.^1 2^ There remains an unmet need for LN treatments that safely and effectively limit disease activity and preserve kidney function.^3^ Standard therapy for class III or IV LN still commonly includes high-dose glucocorticoids plus mycophenolate mofetil (MMF) or cyclophosphamide and is associated with significant toxicity.^4 5^ Targeted LN therapies, belimumab and voclosporin, have recently been approved in some regions/countries;^6^ however, between 60% and 70% of patients receiving treatment did not achieve complete renal response (CRR) at 1 year in the clinical trials leading to regulatory approvals.^7–9^

Anifrolumab is a fully human, IgG1κ monoclonal antibody that targets the type I interferon (IFN) receptor subunit 1.^10^ Anifrolumab is approved in several countries for the treatment of patients with moderate to severe SLE receiving standard therapy^11–14^ based on results of two phase III randomised controlled trials, Treatment of Uncontrolled Lupus via the Interferon Pathway (TULIP)-1 and TULIP-2.^15 16^ Type I IFN-regulated pathways are also involved in the pathogenesis of LN. Studies in a murine model have directly linked IFN and IFN-stimulated genes to pathological and serological changes.^17^ In patients with LN, elevated type I IFN gene signatures (IFNGS) are associated with more active disease and elevated proteinuria;^18^ elevated IFNGS in renal tissues is associated with higher rates of treatment failure.^19^ As the TULIP-1 and TULIP-2 trials excluded patients with active, severe LN,^15 16^ anifrolumab was investigated in this patient population in a separate trial. The randomised, placebo-controlled phase II TULIP-LN (NCT02547922) study^20^ compared the safety and efficacy of two anifrolumab dosing regimens (intensified regimen (IR) and basic regimen (BR)) with placebo added to background standard therapy with glucocorticoids and MMF in patients with active LN over a 2-year period. The primary end point at Week 52, relative improvement from baseline 24-hour urine protein-creatinine ratio (UPCR) in the combined anifrolumab versus placebo groups, was not met.^20^ Pharmacokinetic (PK) data showed that, due to increased drug clearance, a higher anifrolumab dose was required to achieve adequate drug exposure in patients with active LN, compared with patients with non-renal SLE. The anifrolumab IR was numerically superior to placebo for several clinically relevant end points, including more stringent CRR definitions, sustained reduction in oral glucocorticoid dose and markers of disease activity (non-renal clinical SLE Disease Activity Index-2000 (SLEDAI-2K), Physician’s Global Assessment (PGA), Patient’s Global Assessment (PtGA) and complement and antidouble-stranded DNA (anti-dsDNA) antibody levels), while being generally well tolerated. Overall, TULIP-LN study results at Year 1 supported the type I IFN pathway as a potentially promising therapeutic target in LN.^20^ Here, we report the safety, tolerability and exploratory efficacy of 2 years of treatment with anifrolumab added to standard therapy in patients with active LN in the phase II TULIP-LN trial.

## Methods

### Study design

TULIP-LN (NCT02547922) was a phase II, randomised, placebo-controlled double-blind trial. The primary end point, relative difference in mean 24-hour UPCR change from baseline (using geometric mean ratio (GMR) for the combined anifrolumab groups (IR+BR) vs placebo) was assessed at Week 52; these data have been reported.^20^ Second-year efficacy was assessed at Week 104. A safety follow-up period lasted for 12 weeks after the final dose. Trial design is shown in online supplemental figure S1.

10.1136/lupus-2023-000910.supp1 Supplementary data

### Study treatments

At randomisation, included patients were 18–70 years of age with a diagnosis of proliferative class III or IV (±V) LN according to the International Society of Nephrology/Renal Pathology Society 2003 criteria,^21^ proven by biopsy ≤12 weeks prior to signing the consent form or during the screening period. Patients were randomised (1:1:1) to receive anifrolumab IR (900 mg for the first 3 doses, 300 mg thereafter), anifrolumab BR (300 mg, corresponding to SLE dosing^15 16^) or placebo intravenously every 4 weeks through Week 48, with randomisation stratified by IFNGS expression (high vs low) and 24-hour UPCR (≤3.0 vs >3.0 mg/mg) at screening.

To be eligible to continue into Year 2, patients must have completed treatment in Year 1, have not used restricted medications beyond protocol-allowed thresholds and have achieved at least a partial renal response (PRR; estimated glomerular filtration rate (eGFR) ≥60 mL/min/1.73 m^2^ or no confirmed decrease from baseline ≥20% and/or an improvement in 24-hour UPCR (ie, 24-hour UPCR <1.0 mg/mg among patients with a baseline ≤3.0 mg/mg, or 24-hour UPCR ≤3.0 mg/mg and a >50% improvement among patients with a baseline >3.0 mg/mg)) at Week 52. After evaluation at Week 52, eligible patients continued the same blinded study treatment up to and including Week 100.

All patients received standard therapy of oral glucocorticoids and MMF throughout the study. During Year 2, oral glucocorticoid dosage (prednisone/equivalent) was required to be ≤7.5 mg/day by Week 60 and ≤5.0 mg/day by Week 80. After Week 60, MMF was required to be ≤2.0 g/day or the Week 52 dosage or below, whichever was lower. Stable oral glucocorticoid and MMF dosages were required during Weeks 92–104. Additional standard therapy protocol details are in the online supplemental methods.

### Discontinuation criteria

At any time, study treatment discontinuation was required if patients experienced LN worsening (an LN-related, confirmed eGFR decrease >30% from baseline to <60 mL/min/1.73 m^2^ in two independent samples, an increase in renal or extrarenal lupus activity requiring prohibited immunosuppressive treatment (cyclophosphamide, rituximab, belimumab)), or if oral glucocorticoid tapering targets were not met.

### Study end points

Second-year end points were exploratory, focused on characterising safety and tolerability of long-term anifrolumab use; full descriptions can be found in online supplemental methods. Safety assessments, presented in this manuscript for study Year 1 and Year 2, included adverse events (AEs), laboratory assessments and vital signs.

Efficacy end points included the relative difference in mean change from baseline to Week 104 in 24-hour UPCR in the combined anifrolumab versus placebo groups; the proportion of patients at Week 104 attaining a CRR (24-hour UPCR ≤0.7 mg/mg), PRR (defined previously), alternative CRR (aCRR, defined as a CRR with inactive urinary sediment (<10 red blood cells per high power field)), sustained oral glucocorticoid taper and CRR with sustained oral glucocorticoid taper. Prespecified exploratory end points also included cumulative oral glucocorticoid dose; mean change from baseline in non-renal SLEDAI-2K,^22^ PGA,^23^ PtGA^24^ and lupus serologies (anti-dsDNA antibodies, complement C3/C4) and the immunogenicity, PK and pharmacodynamic (PD) profiles of anifrolumab. PD neutralisation was measured as the median percentage change of baseline 21-gene type I IFNGS (21-IFNGS), as described previously.^10 20 25 26^ Post hoc efficacy analyses included cumulative proteinuria, adjusted geometric mean eGFR, the proportion of patients achieving CRR with UPCR ≤0.5 mg/mg (CRR_0.5_) and the probability of achieving sustained CRR_0.5_ response through Week 104.

### Statistical analysis

Safety and efficacy analyses were conducted using the full analysis set population, that is, all randomised patients who received ≥1 dose of treatment. Year 2 safety data, presented based on the number of patients who continued treatment in Year 2, were split from Year 1 post hoc. As the Italian Medicines Agency and the France Ethics Committee did not agree to a protocol amendment that included changes to cut-off values for renal function and proteinuria components of the renal response end points (UPCR ≤0.7 mg/mg and eGFR ≥60 mL/min/1.73 m^2^ and ≤20% decrease), 13 patients enrolled in Italy and France were excluded from second-year analyses of composite renal end points.

All end points were exploratory and not formally tested. Safety was analysed descriptively. Continuous variables were analysed using a mixed model for repeated measures (MMRM), controlling for stratification factors and baseline values, with separate MMRMs fitted for Year 1 and Year 2. Missing data for continuous end points were modelled under the missing-at-random assumption within the MMRM model. eGFR and 24-hour UPCR data were log-transformed for analysis. Binary end points, responder rates and 95% CIs were calculated using a stratified Cochran-Mantel-Haenszel approach, controlling for stratification factors. Cumulative oral glucocorticoid dose was evaluated with summary statistics by treatment group. All analyses were performed with Statistical Analysis System (SAS Institute, Inc., Cary, North Carolina, USA), V.9.3 or higher.

### Patient and public involvement

No patients or members of the public were involved in the design of this trial.

## Results

### Trial population

**Figure 1:**
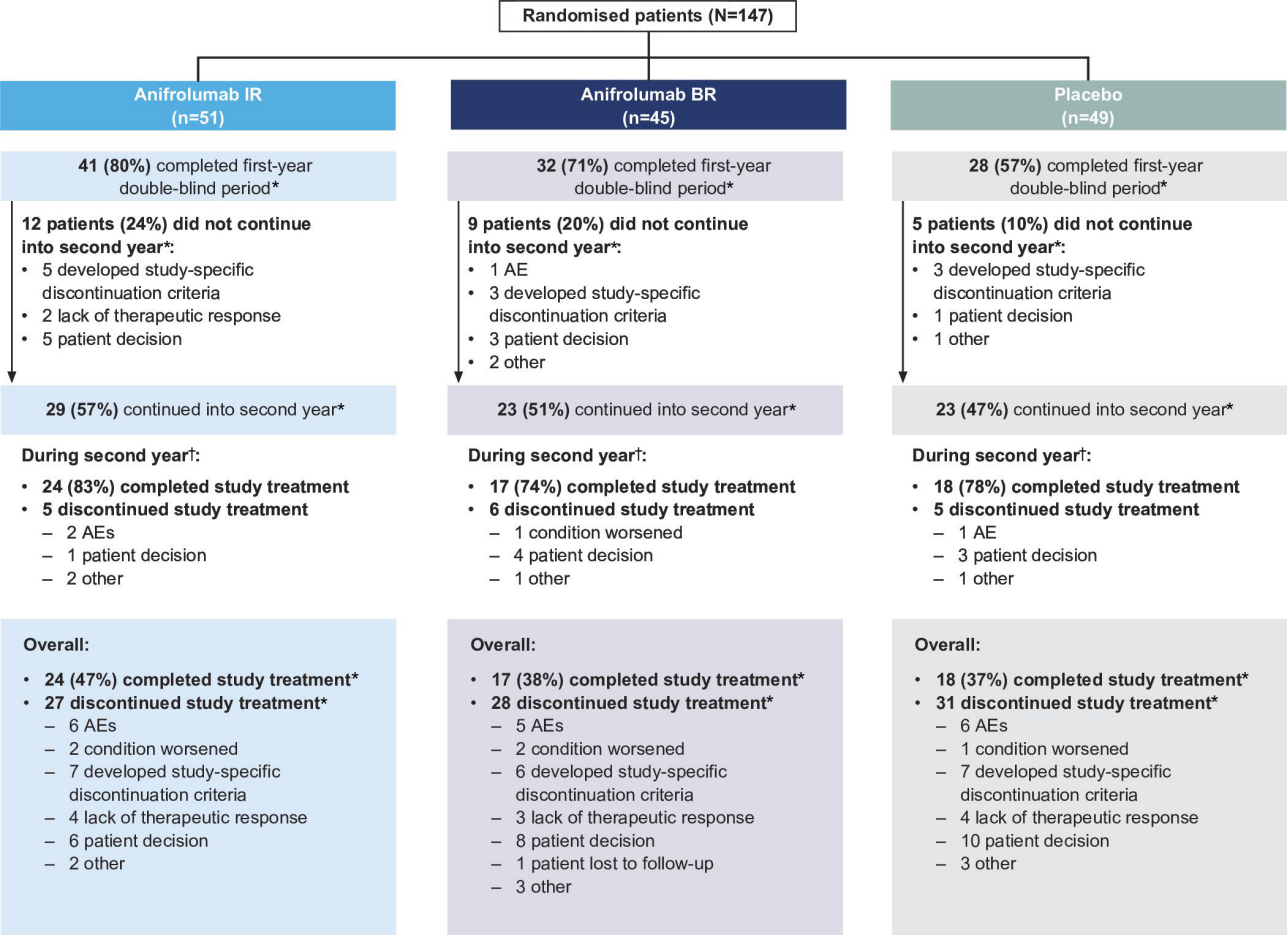
Patient disposition for the first and second years of the TULIP-LN study. *Percentages calculated using the full analysis set. ^†^Percentages calculated using the second-year population (patients who continued into the second year). AE, adverse event; BR, basic regimen; IR, intensified regimen; TULIP, Treatment of Uncontrolled Lupus via the Interferon Pathway.

Of the 147 patients randomised to study Year 1 between November 2015 and November 2018, 145 patients received ≥1 dose of treatment (anifrolumab IR: n=51, anifrolumab BR: n=45 and placebo: n=49). Patient disposition for the 2-year period is shown in figure 1.

Through Week 52, 41 patients (80%) receiving anifrolumab IR, 32 (71%) receiving anifrolumab BR and 28 (57%) receiving placebo completed study treatment. Of all patients dosed, 29 (57%) in the anifrolumab IR group, 23 (51%) in the anifrolumab BR group and 23 (47%) in the placebo group continued into Year 2. Eleven patients who completed Year 1 did not continue to Year 2 because they did not meet protocol-specified continuation criteria (ie, achieving at least PRR and meeting glucocorticoid tapering targets); 15 did not continue due to patient decision (n=9), lack of therapeutic response (n=2), AE (n=1) or other reasons (n=3).

Among patients who rolled over into Year 2, 24 (83%) in the anifrolumab IR group, 17 (74%) in the anifrolumab BR group and 18 (78%) in the placebo group completed study treatment (figure 1). The most frequently recorded reason for treatment discontinuation was patient decision, which was more frequent with anifrolumab BR (n=4; 17%) and placebo (n=3; 13%) than anifrolumab IR (n=1; 3%). Discontinuation rates varied somewhat between regions, but no apparent imbalance was noted.

Demographics, baseline disease characteristics and SLE-related medication use are shown in table 1; disease characteristics at end of Year 1 of patients who continued into Year 2 are shown in online supplemental table S1.

**Table 1:** Patient demographics and disease characteristics of the overall and second-year population

		Anifrolumab IR		Anifrolumab BR		Placebo	
		Overall (n=51)	Second-year population (n=29)	Overall (n=45)	Second-year population (n=23)	Overall (n=49)	Second-year population (n=23)
**Patient demographics**							
Age, years	Median (range)	35 (18, 65)	39 (18, 59)	34 (19, 67)	37 (19, 67)	32 (18, 58)	35 (22, 58)
Sex	Female, n (%)	45 (88.2)	29 (100)	37 (82.2)	22 (95.7)	38 (77.6)	15 (65.2)
Weight	Mean (SD), kg	67.7 (16.8)	61.0 (12.9)	62.7 (12.3)	59.2 (9.1)	65.6 (13.3)	68.6 (13.2)
BMI	Mean (SD)	26.0 (5.9)	24.4 (4.5)	24.0 (3.8)	23.7 (3.9)	24.5 (3.9)	24.6 (3.9)
	>28 kg/m^2^, n (%)	16 (31.4)	6 (20.7)	7 (15.6)	3 (13.0)	9 (18.4)	5 (21.7)
Race, n (%)	White	25 (49.0)	13 (44.8)	17 (37.8)	7 (30.4)	24 (49.0)	15 (65.2)
	Black/African-American	4 (7.8)	1 (3.4)	2 (4.4)	0	1 (2.0)	0
	Asian	7 (13.7)	5 (17.2)	11 (24.4)	7 (30.4)	10 (20.4)	4 (17.4)
	Native Hawaiian/Pacific Islander	0	0	1 (2.2)	1 (4.3)	0	0
	American Indian/Alaska Native	1 (2.0)	1 (3.4)	3 (6.7)	1 (4.3)	0	0
	Other	14 (27.5)	9 (31.0)	11 (24.4)	7 (30.4)	14 (28.6)	4 (17.4)
Ethnicity, n (%)	Hispanic or Latino	23 (45.1)	14 (48.3)	22 (48.9)	10 (43.5)	20 (40.8)	8 (34.8)
	Not Hispanic or Latino	28 (54.9)	15 (51.7)	23 (51.1)	13 (56.5)	29 (59.2)	15 (65.2)
Geographic region, n (%)	Asia Pacific	8 (15.7)	5 (17.2)	10 (22.2)	7 (30.4)	9 (18.4)	4 (17.4)
	Europe	16 (31.4)	10 (34.5)	10 (22.2)	5 (21.7)	15 (30.6)	9 (39.1)
	Latin America	20 (39.2)	12 (41.4)	14 (31.1)	7 (30.4)	16 (32.7)	7 (30.4)
	North America	7 (13.7)	2 (6.9)	11 (24.4)	4 (17.4)	9 (18.4)	3 (13.0)
**Baseline disease characteristics**							
Renal biopsy result at screening, n (%)	Class III	10 (19.6)	8 (27.6)	7 (15.6)	4 (17.4)	6 (12.2)	3 (13.0)
	Class III+V	4 (7.8)	3 (10.3)	7 (15.6)	3 (13.0)	5 (10.2)	2 (8.7)
	Class IV	27 (52.9)	14 (48.3)	26 (57.8)	14 (60.9)	30 (61.2)	14 (60.9)
	Class IV+V	10 (19.6)	4 (13.8)	5 (11.1)	2 (8.7)	8 (16.3)	4 (17.4)
24-hour UPCR, mg/mg	Mean (SD)	2.9 (1.9)	2.7 (1.8)	3.4 (2.5)	3.0 (2.6)	3.7 (3.2)	3.3 (2.0)
	>3.0, n (%)	17 (33.3)	7 (24.1)	19 (42.2)	7 (30.4)	23 (46.9)	11 (47.8)
eGFR*, mL/min/1.73 m^2^	Mean (SD)	94.4 (43.2)	94.3 (41.7)	100.2 (46.8)	97.2 (33.7)	87.3 (35.4)	75.1 (28.5)
	≥60, n (%)	38 (74.5)	23 (79.3)	35 (77.8)	18 (78.3)	39 (79.6)	17 (73.9)
SLEDAI-2K score	Mean (SD)	11.0 (5.0)	10.2 (4.3)	10.4 (4.6)	9.5 (4.3)	11.3 (4.4)	10.4 (4.7)
Non-renal SLEDAI-2K score	Mean (SD)	4.2 (2.7)	4.4 (2.6)	5.2 (3.4)	4.5 (3.5)	4.7 (2.3)	4.3 (2.7)
IFNGS status	High, n (%)	47 (92.2)	27 (93.1)	44 (97.8)	23 (100)	46 (93.9)	20 (87.0)
Serology, n (%)	ANA positive†	46 (90.2)	28 (96.6)	44 (97.8)	23 (100)	49 (100)	23 (100)
	Anti-dsDNA positive‡	39 (76.5)	22 (75.9)	37 (82.2)	19 (82.6)	39 (79.6)	19 (82.6)
	Low C3§	27 (52.9)	16 (55.2)	30 (66.7)	15 (65.2)	42 (85.7)	18 (78.3)
	Low C4§	14 (27.5)	9 (31.0)	10 (22.2)	6 (26.1)	20 (40.8)	5 (21.7)
**Baseline treatments**							
Oral glucocorticoids¶	Yes, n (%)	51 (100)	29 (100)	43 (95.6)	23 (100.0)	48 (98.0)	23 (100)
	Dosage, mean (SD), mg/day	23.2 (10.9)	23.1 (11.1)	21.9 (10.4)	23.4 (10.2)	21.9 (11.2)	22.4 (11.8)
MMF/MPA before randomisation	Yes, n (%)	36 (70.6)	21 (72.4)	36 (80.0)	17 (73.9)	33 (67.3)	16 (69.6)
MMF dose at randomisation	Mean (SD), g/day	1.8 (0.5)	1.8 (0.4)	1.8 (0.6)	1.8 (0.6)	1.8 (0.5)	1.8 (0.4)
Concomitant ACEI/ARB treatment, n (%)		36 (70.6)	21 (72.4)	27 (60.0)	11 (47.8)	33 (67.3)	16 (69.6)
Antimalarials, n (%)		26 (51.0)	17 (58.6)	31 (68.9)	16 (69.6)	35 (71.4)	16 (69.6)

Baseline is defined as the last measurement prior to randomisation and dose administration on Day 1 of Year 1.

*eGFR is calculated using the MDRD formula.

†ANA positive was defined as a titre ≥1:40.

‡Anti-dsDNA positive was defined as an anti-dsDNA level above the assay cut-off for positive.

§Low complement level at baseline was defined as a complement level below lower limit of normal.

¶Baseline oral glucocorticoid dosage is defined as the maximum daily dose of prednisone or equivalent taken between Day 1 and Day 7, inclusive.

ACEI, angiotensin-converting enzyme inhibitors; ANA, antinuclear antibodies; anti-dsDNA, antidouble-stranded DNA; ARB, angiotensin receptor blockers; BMI, body mass index; BR, basic regimen; C3, complement 3; C4, complement 4; eGFR, estimated glomerular filtration rate; IFNGS, interferon gene signature; IR, intensified regimen; MDRD, Modification of Diet in Renal Disease; MMF, mycophenolate mofetil; MPA, mycophenolic acid; SLEDAI-2K, Systemic Lupus Erythematosus Disease Activity Index 2000; UPCR, urine protein-creatinine ratio.

#### Safety and tolerability

##### Exposure

A greater proportion of patients in the anifrolumab IR group received all 26 infusions up to Week 100, compared with the anifrolumab BR and placebo groups (39.2% vs 22.2% and 26.5%). Thus, total exposure was greatest in the anifrolumab IR group (73.3 patient-years (PY)) versus anifrolumab BR (58.4 PY) and placebo (57.3 PY) groups.

##### Adverse events

AEs during treatment are shown by study year in table 2 (overall non-serious AEs (SAEs) in online supplemental table S2). Rates of AEs and SAEs per 100 PY were lower in Year 2 than Year 1 (AEs, Year 1 vs Year 2, anifrolumab IR: 99.5 vs 74.9; BR: 108.7 vs 93.6 and placebo: 119.0 vs 72.0; SAEs, anifrolumab IR: 15.1 vs 7.5; BR: 23.3 vs 10.4 and placebo: 22.1 vs 4.8) (table 2). Over the study period, percentages of patients discontinuing due to AEs were 11.8%, 11.1% and 12.2% in the anifrolumab IR, BR and placebo groups, respectively. There were no deaths during treatment. One fatal vascular neurological AE, considered by the investigator to be unrelated to study treatment, occurred in the anifrolumab BR group during follow-up.

**Table 2:** AEs during treatment by study year

Patients, n (%)	Year 1						Year 2					
	Anifrolumab IR (n=51)		Anifrolumab BR (n=45)		Placebo (n=49)		Anifrolumab IR (n=29)		Anifrolumab BR (n=23)		Placebo (n=23)	
Exposure, years	46.22		38.62		36.14		26.70		19.23		20.82	
	n (%) of patients	EAIR per 100 PY*	n (%) of patients	EAIR per 100 PY*	n (%) of patients	EAIR per 100 PY*	n (%) of patients	EAIR per 100 PY*	n (%) of patients	EAIR per 100 PY*	n (%) of patients	EAIR per 100 PY*
Any AE	46 (90.2)	99.5	42 (93.3)	108.7	43 (87.8)	119.0	20 (69.0)	74.9	18 (78.3)	93.6	15 (65.2)	72.0
Any AE with outcome of death	0	0	0	0	0	0	0	0	0	0	0	0
Any SAE including outcome of death	7 (13.7)	15.1	9 (20.0)	23.3	8 (16.3)	22.1	2 (6.9)	7.5	2 (8.7)	10.4	1 (4.3)	4.8
AEs of special interest	7 (13.7)	15.1	9 (20.0)	23.3	7 (14.3)	19.4	5 (17.2)	18.7	3 (13.0)	15.6	1 (4.3)	4.8
Non-opportunistic serious infections†	0	0	0	0	2 (4.1)	5.5	1 (3.4)	3.7	0	0	1 (4.3)	4.8
Opportunistic infections‡	0	0	1 (2.2)	2.6	1 (2.0)	2.8	0	0	0	0	0	0
Anaphylaxis	0	0	0	0	0	0	0	0	0	0	0	0
Malignancy	0	0	0	0	0	0	1 (3.4)	3.7	0	0	0	0
Herpes zoster	6 (11.8)	13.0	8 (17.8)	20.7	4 (8.2)	11.1	1 (3.4)	3.7	1 (4.3)	5.2	0	0
Latent tuberculosis	0	0	0	0	0	0	0	0	0	0	0	0
Influenza	1 (2.0)	2.2	0	0	1 (2.0)	2.8	3 (10.3)	11.2	2 (8.7)	10.4	0	0
Vasculitis (non-SLE)	0	0	0	0	0	0	0	0	0	0	0	0
Major adverse cardiovascular events according to the CV-EAC	0	0	0	0	1 (2.0)	2.8	0	0	0	0	0	0

AEs are coded using MedDRA V.22.1. Percentages are based on the 145 patients who received ≥1 dose of anifrolumab or placebo. An AE during treatment in Year 1 is defined as an AE with a date and time of onset ≥date and time of first dose of investigational product and ≤date of last dose of investigational product+28 days or date of end of Year 1 period for that participant. An AE during treatment in Year 2 is defined as an AE with a date of onset ≥end of Year 1 period for that participant and ≤date of last dose of investigational product+28 days. AEs reported in the study by Jayne *et al* included all safety data collected up until the data cut-off following the last patient completing the Week 52 visit, which may have included AEs during Year 2 for some patients.

*Exposure-adjusted incidence rate per 100 PY was calculated by dividing the number of patients experiencing an event by the total exposure time in days for all participants in the analysis set, multiplied by 365.25, multiplied by 100.

†Excludes tuberculosis and influenza.

‡Excludes herpes zoster.

AE, adverse event; BR, basic regimen; CV-EAC, Cardiovascular Event Adjudication Committee; EAIR, exposure-adjusted incidence rate; IR, intensified regimen; MedDRA, Medical Dictionary for Regulatory Activities; PY, patient-years; SAE, serious adverse event.

On 11 March 2020, when World Health Organization (WHO) declared COVID-19 a pandemic,^27^ 12 patients remained on treatment and 5 patients were in follow-up. No COVID-19–related AEs, including positive COVID-19 tests, were reported.

Among AEs of special interest (AESIs), herpes zoster (HZ) and influenza were reported more frequently in the anifrolumab groups versus placebo (table 2). In Year 1, the event rates of HZ per 100 PY were 13.0 (n=6), 20.7 (n=8) and 11.1 (n=4) with anifrolumab IR, anifrolumab BR and placebo, respectively. In Year 2, there was one HZ case in the anifrolumab IR group, one in the anifrolumab BR group and none in the placebo group. Of the 16 HZ cases in the anifrolumab groups, 6 were SAEs and all were cutaneous (13 localised, 3 multidermatomal and none were ophthalmic). HZ events tended to occur early in the trial and resolved with conventional treatment. Incidences of other AESIs during treatment were low across groups. In Year 2, there was each one case of non-opportunistic serious infection in the anifrolumab IR and placebo groups, and no opportunistic infections. There were five cases of influenza in Year 2 and two cases in Year 1. There were no cases of active tuberculosis or anaphylaxis; the one malignancy case (endometrial adenocarcinoma) was in the anifrolumab IR group (table 2).

Overall, anti-drug antibodies were detected in seven patients (anifrolumab IR: n=2; BR: n=3; placebo: n=2). Only one patient, who was in the placebo group, was positive after baseline.

#### Efficacy

##### Complete renal response

**Figure 2:**
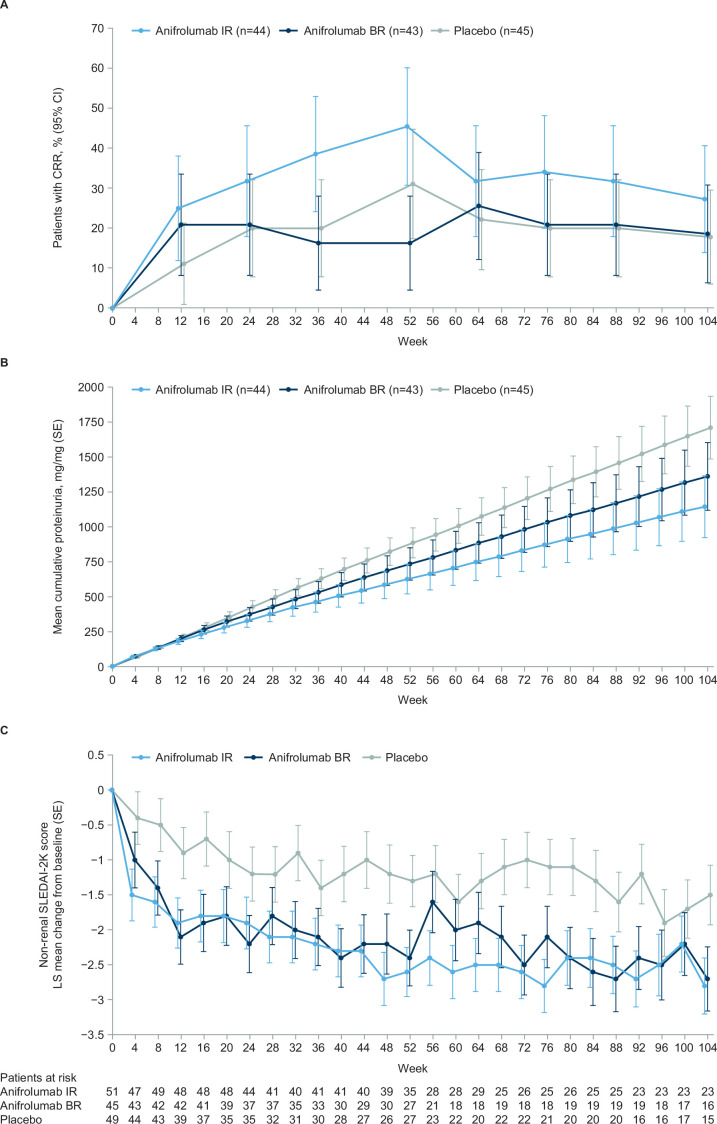
Efficacy end points over time. Analyses were conducted excluding patients from France and Italy. All data after discontinuation were excluded from the analysis. (A) CRR required 24-hour UPCR ≤0.7 mg/mg, eGFR ≥60 mL/min/1.73 m^2^ or no decrease ≥20% from baseline, no treatment discontinuation and no use of restricted medications. The response rates were calculated with a weighted Cochran-Mantel-Haenszel controlling for stratification factors; percentages are based on the number of patients in the analysis, so the denominator remained the same each week (anifrolumab IR: n=44; anifrolumab BR: n=43; placebo: n=45). Non-responder imputation was applied in case of missing data in any of the CRR components. (B) Mean cumulative proteinuria (area under the curve in UPCR standardised by the expected follow-up time) was assessed using analysis of covariance controlling for baseline UPCR and stratification factors. Error bars represent SE. (C) Non-renal SLEDAI-2K change from baseline is expressed as least squares means which were calculated using a mixed model for repeated measures, controlling for stratification factors. Missing data for continuous end points were modelled under the missing-at-random assumption within the mixed model for repeated measures model. BR, basic regimen, CRR, complete renal response; eGFR, estimated glomerular filtration rate; IR, intensified regimen; LS, least squares; n, number of subjects in the treatment group; SLEDAI-2K, SLE Disease Activity Index 2000; UPCR, urine protein-creatinine ratio.

At Week 104, a numerically greater proportion of anifrolumab IR patients attained CRR versus placebo (27.3% vs 17.8%; treatment difference [Δ]=9.5% (95% CI −8.4 to 27.4)), whereas similar proportions of patients attained CRR in the anifrolumab BR and placebo groups (18.6% vs 17.8; Δ=0.8% (95% CI −16.2 to 17.8)) (table 3). Proportions of patients achieving aCRR at Week 104 were similar across groups (IR: 15.9%; BR: 16.3% and placebo: 17.8%) (table 3). A numerically greater proportion of anifrolumab IR patients attained CRR versus other treatments over the study period (figure 2A).

**Table 3:** Efficacy summary at Week 104

End points, week 104		Anifrolumab IR (n=51)	Anifrolumab BR (n=45)	Placebo (n=49)
CRR*	n/N (%)	12/44 (27.3)	8/43 (18.6)	8/45 (17.8)
	Difference, % (95% CI)	9.5 (−8.4 to 27.4)	0.8 (−16.2 to 17.8)	–
At least a PRR*	n/N (%)	15/44 (34.1)	10/43 (23.3)	8/45 (17.8)
	Difference, % (95% CI)	16.3 (−2.1 to 34.7)	5.5 (−12.1 to 23.0)	–
CRR_0.5_ (requiring UPCR ≤0.5 mg/mg)*†	n/N (%)	9/44 (20.5)	4/43 (9.3)	5/45 (11.1)
	Difference, % (95% CI)	9.3 (−6.7 to 25.4)	−1.8 (−16.1 to 12.5)	–
**aCRR** (requiring inactive urinary sediment)*	n/N (%)	7/44 (15.9)	7/43 (16.3)	8/45 (17.8)
	Difference, % (95% CI)	−1.9 (−18.4 to 14.6)	−1.5 (−18.1 to 15.1)	–
Sustained oral glucocorticoid dosage reduction (≤5.0 mg/day, Weeks 80–104)‡	n/N (%)	13/36 (36.1)	7/31 (22.6)	10/33 (30.3)
	Difference, % (95% CI)	5.8 (−16.7 to 28.3)	−7.7 (−29.9 to 14.5)	–
CRR with sustained oral glucocorticoid dosage reduction	n/N (%)	11/44 (25.0)	8/43 (18.6)	8/45 (17.8)
	Difference, % (95% CI)	7.2 (−10.4 to 24.9)	0.8 (−16.2 to 17.8)	–

No end points met nominal significance for either anifrolumab group versus placebo at week 104. Response rates, differences between groups and associated 95% CIs were calculated with a weighted Cochran-Mantel-Haenszel method controlling for stratification factors, excluding 13 patients from France and Italy (anifrolumab IR: n=7, BR: n=2 and placebo: n=4). Differences between anifrolumab and placebo groups were calculated in percentage points (the percentage in the anifrolumab group minus the placebo group).

*Patients from France and Italy were excluded.

†Analysed post hoc.

‡Analysed in patients with baseline oral glucocorticoid dosage ≥20 mg/day.

aCRR, alternative CRR; BR, basic regimen; CRR_0.5_, CRR with UPCR ≤0.5mg/mg; CRR, complete renal response; N, number of patients included in the analysis; n, number of patients meeting the criteria for a response; PRR, partial renal response; UPCR, urine protein-creatinine ratio.

Proportions of patients who attained the individual components of the CRR and aCRR criteria at Week 104 are shown in online supplemental table S3. There were numerical trends towards greater improvements in eGFR with anifrolumab IR versus placebo throughout the 2-year period (online supplemental figure S2).

CRR_0.5_ at Week 104 was attained by a numerically greater proportion of patients with anifrolumab IR versus placebo (20.5% vs 11.1%; Δ=9.3% (95% CI −6.7 to 25.4)), but not with anifrolumab BR versus placebo (9.3% vs 11.1%; Δ=−1.8% (95% CI −16.1 to 12.5)) (table 3). A numerically greater proportion of anifrolumab IR patients attained CRR_0.5_ versus other treatments over the study period (online supplemental figure S3). Patients receiving anifrolumab IR were more likely to achieve a CRR_0.5_ that was sustained through Week 104 vs patients receiving placebo (HR 1.9; 95% CI 0.6 to 7.2); this difference was not observed for patients receiving anifrolumab BR (HR 0.9; 95% CI 0.2 to 3.7) (online supplemental figure S4).

The proportion of patients who attained at least a PRR at Week 104 was almost twofold greater with anifrolumab IR versus placebo (34.1% vs 17.8%; Δ=16.3% (95% CI −2.1 to 34.7)), which was not observed with anifrolumab BR (23.3% vs 17.8%; Δ=5.5% (95% CI −12.1 to 23.0)) (table 3).

##### Oral glucocorticoid use

To Week 104, mean cumulative oral glucocorticoid doses in the anifrolumab IR and BR groups were 14.2% and 16.7% lower than in the placebo group, respectively (online supplemental figure S5). The mean oral glucocorticoid daily dose (SD) decreased from baseline to week 104 across groups (anifrolumab IR: −20.1 (13.3) mg/day, BR: −20.7 (11.7) mg/day and placebo: −19.9 (12.2) mg/day). Sustained oral glucocorticoid dosage reduction from ≥20 to ≤5.0 mg/day (prednisone/equivalent) through Weeks 80–104 was attained by 36.1% of the anifrolumab IR group, 30.3% of the placebo group (Δ=5.8% (95% CI −16.7 to 28.3)) and 22.6% of the anifrolumab BR group (Δ=−7.7% vs placebo (95% CI −29.9 to 14.5)) (table 3). CRR and sustained oral glucocorticoid tapers were achieved by 25.0% of patients with anifrolumab IR, 17.8% with placebo (Δ=7.2% (95% CI −10.4 to 24.9)) and 18.6% with anifrolumab BR (Δ=0.8% vs placebo (95% CI −16.2 to 17.8)) (table 3).

##### Relative improvement in baseline 24-hour UPCR

Mean 24-hour UPCR improved 83% from baseline to Week 104 with anifrolumab IR, 80% with anifrolumab BR and 80% with placebo (online supplemental figure S6). GMR was 0.9 for anifrolumab IR versus placebo (95% CI 0.4 to 1.7) and 1.0 for anifrolumab BR versus placebo (95% CI 0.5 to 2.0), where GMR <1 favours anifrolumab. Cumulative UPCR (area under the curve for proteinuria) throughout treatment is shown in figure 2B. At Week 104, mean cumulative proteinuria with anifrolumab IR was one-third less than with placebo.

##### Non-renal SLE disease activity

Mean non-renal SLEDAI-2K score decreased from baseline to a greater extent in both anifrolumab groups versus placebo from as early as Week 4 through to Week 104 (least squares mean (SE) change from baseline to Week 104, anifrolumab IR: −2.8 (0.4); anifrolumab BR: −2.7 (0.5) and placebo: −1.5 (0.4)) (figure 2C). Similarly, PGA and PtGA scores decreased across all groups over the study but decreased to the greatest extent in the anifrolumab IR group (online supplemental figure S7).

##### Disease serological activity

Levels of serological markers varied over time (online supplemental figure S8). Improvements from baseline in anti-dsDNA antibody titres and C3 levels were observed up to Week 104 in patients in both anifrolumab groups, with the greater improvement in the anifrolumab IR group; in the placebo group, serological markers varied non-directionally throughout the study. There were no obvious trends in C4 level changes from baseline for any group over the study period (online supplemental figure S8).

### Pharmacokinetics and pharmacodynamics

C_trough_ concentrations increased steadily in both groups through Week 104 to 35.2 µg/mL (IR) and 32.2 μg/mL (BR). During the first 52 weeks, interindividual variability in anifrolumab serum exposure was greater with anifrolumab BR versus anifrolumab IR; this difference converged and stabilised during Year 2 (online supplemental figure S9).

PD neutralisation of IFNGS over the study in 137 IFNGS-high patients is shown in online supplemental figure 10. The median 21-IFNGS neutralisation was >80% across all visits with anifrolumab IR, but only from Week 52 onwards with anifrolumab BR. At Week 104, the median percent neutralisation of the baseline 21-IFNGS was similar between anifrolumab groups (IR: 90.1% and BR: 91.8%). No neutralisation was observed in the placebo group at any timepoint.

## Discussion

Here, we report the second-year analyses of the phase II TULIP-LN trial, investigating the safety and efficacy of two dosing regimens of anifrolumab added to standard therapy, in patients with active LN. Overall, the second-year findings are consistent with the Year 1 results.^20^ Only patients who completed Year 1 study treatment achieved at least PRR and met the protocol-required glucocorticoid tapering targets and safety-related criteria were eligible to enter Year 2. The number of patients entering the second year of the trial was therefore reduced. This may impact data interpretation by driving non-response for binary end points and hindering detection of treatment differences for continuous end points, where data were assumed to be missing at random; however, this approach allowed comparison of outcomes in patients with at least PRR on placebo plus standard therapy rather than a combination of responders and non-responders. Trends at Week 104 support use of an intensified dosing regimen of anifrolumab in patients with active LN relative to non-renal SLE; indeed, anifrolumab IR, but not anifrolumab BR, treatment was associated with numerically beneficial treatment responses across a range of clinically meaningful outcome measures, including CRR with sustained oral glucocorticoid taper, and improvement in overall disease activity and serological activity over placebo.

As previously reported,^20^ the primary end point of TULIP-LN was not met. This was likely because improvement in 24-hour UPCR in the combined anifrolumab group from baseline to Week 52 was negatively impacted by the suboptimal exposure with BR dosing. At Week 104, 24-hour UPCR decreased substantially from baseline (by ≥80%) across all groups. It is worth noting that 24-hour UPCR improvement may have been overestimated for all treatment groups, partly owing to stringent discontinuation criteria leading to a high rate of treatment discontinuation and, consequently, missing data. Missing data were modelled through the MMRM analysis under a missing-at-random assumption, confounding the model-estimated treatment effect. This phenomenon was probably greatest in the placebo and anifrolumab BR groups, where discontinuation rates were higher than in the anifrolumab IR group. These confounding factors were partly overcome by assessing cumulative UPCR, which represents proteinuria over the entire treatment duration. Cumulative UPCR showed that proteinuria was lower in both anifrolumab groups than the placebo group; at Week 104, mean cumulative proteinuria in the anifrolumab IR group was one-third less than in the placebo group. This is important, as proteinuria reduction signals decreased inflammation in the kidney, improved kidney function and is associated with reduced risk of end-stage kidney disease.^28–30^

Obtaining 24-hour UPCR ≤0.5 mg/mg for prolonged periods of time is associated with good prognoses and reduced likelihood of end-stage kidney disease and is a recommended LN treatment goal.^4 31^ Here, CRR_0.5_ responses, which capture UPCR ≤0.5 mg/mg, also tended to be sustained throughout the 2-year trial duration with anifrolumab IR. Although definitions vary among studies, attainment of CRR has been associated with patient survival^32–34^ and is a widely used end point in LN clinical trials.^35 36^ Over the 2-year period, anifrolumab IR treatment was associated with consistently greater CRR rates versus placebo, both for definitions requiring 24-hour UPCR ≤0.5 and ≤0.7 mg/mg. CRR responses occurred early in the trial, a valuable attribute for an LN treatment.^37^

Oral glucocorticoid reduction with anifrolumab is also an important feature, as tapering is a recommended aim of SLE as well as LN treatment^4 5^ due to toxicity and organ damage associated with prolonged, high-dose glucocorticoid use.^4 5^ Here, the cumulative oral glucocorticoid dose was ∼15% lower in both anifrolumab groups than in the placebo group. The proportions of patients who had sustained oral glucocorticoid tapering to ≤5.0 mg/day—a stringent threshold, considering the typical tapering goal is ≤7.5 mg/day^4 5^—was also numerically greater with anifrolumab IR versus placebo.

After Week 52, anifrolumab serum exposures and IFNGS neutralisation were similar between anifrolumab BR and IR; however, response rates with anifrolumab BR were consistently lower than or similar to placebo across end points. This suggests the first 3 ‘pulse’ doses of anifrolumab 900 mg in the IR group were crucial to the initial neutralisation, and this was maintained by the 300 mg doses.

Anifrolumab was generally well tolerated throughout the study. Similar to the results of the primary analysis at Week 52,^20^ the safety profile of anifrolumab for patients with LN was generally consistent with the safety profile in trials of anifrolumab in patients with SLE without active renal disease.^20 38^ Most AEs were not serious and did not lead to treatment discontinuation, and there were two cases of HZ in the second year of study.^20^

This analysis had several limitations. First, although the study was designed and powered to evaluate efficacy and safety in the combined anifrolumab IR and BR groups versus placebo, the primary analysis suggested that a higher dose was needed in patients with LN relative to non-renal SLE. Therefore, each dosing group was compared with placebo separately. Further exploration in larger numbers of patients with active LN would be required to draw conclusions about the long-term efficacy and safety of anifrolumab in this patient population. The high rate of discontinuation also impacted the numbers of patients in each treatment arm who completed the study. It is worth noting that patients receiving anifrolumab IR were less likely to discontinue, with 47% completing all planned doses to Week 104, compared with 38% and 37% in the anifrolumab BR and placebo groups, respectively. The strict protocol-defined discontinuation criteria applied, and contributed to discontinuation, in all treatment groups. Most discontinuations in the anifrolumab IR group were required by these study-specific discontinuation criteria, including predefined glucocorticoid taper targets, requirements for renal disease improvement or use of prohibited medications, which may be less reflective of clinical practice.^4^ The most frequent reason for discontinuation in the anifrolumab BR and placebo groups was patient decision (18%–20% vs 12% with anifrolumab IR), which may be more applicable to clinical settings; indeed, several studies have shown that patients who feel they are not benefitting from a drug, or who have concerns about its safety, are likely to have poor therapy compliance.^39 40^ Although discontinuation rates varied somewhat between regions, no apparent imbalance was noted; the numbers of patients discontinuing treatment in individual countries or sites were too small to draw meaningful conclusions.

Another potential limitation of this study was the confounding impact of background standard therapies. Such confounding effects are commonplace in LN trials, where use of high-dose background steroids may mask effects of new biologics, and high placebo responses can lead to small effect sizes, necessitating large patient cohorts.^41^ However, this may have been partly overcome by the stringent protocol-specified tapering requirements.

The second-year TULIP-LN study results are generally consistent with those reported in the primary analysis,^20^ with numerical treatment differences favouring anifrolumab IR over placebo for several clinically relevant end points suggesting that anifrolumab IR was required to obtain clinical efficacy. These treatment differences were generally sustained over 2 years. Compared with the anifrolumab IR group, patients treated with anifrolumab BR had lower serum exposures and more variable PD neutralisation during Year 1, with responses similar to or lower than placebo across clinical end points for the entire 2-year period. As such, these results support the primary analysis conclusion that anifrolumab IR is the dosing regimen of choice for future clinical investigations in a larger population of patients with active LN. A phase III trial of anifrolumab in patients with active, proliferative LN has been initiated (NCT05138133).

### Trials mentioned

IRIS: currently recruiting, ‘A Multicentre Randomised Double-Blind Placebo Controlled Phase III Study to Evaluate the Efficacy and Safety of Anifrolumab in Adult Patients With Active Proliferative Lupus Nephritis’ (NCT05138133).

TULIP-1: completed, ‘A Multicentre, Randomised, Double-blind, Placebo-controlled, Phase III Study Evaluating the Efficacy and Safety of Two Doses of Anifrolumab in Adult Subjects With Active Systemic Lupus Erythematosus’ (NCT02446912).

TULIP-2: completed, ‘A Multicentre, Randomised, Double-blind, Placebo-controlled, Phase III Study Evaluating the Efficacy and Safety of Anifrolumab in Adult Subjects With Active Systemic Lupus Erythematosus’ (NCT02446899).

TULIP-LN: completed, ‘A Multicentre, Randomised, Double-blind, Placebo-controlled, Phase II Study Evaluating the Efficacy and Safety of Anifrolumab in Adult Subjects With Active Proliferative Lupus Nephritis’ (NCT02547922).

## Data Availability

Data are available on reasonable request. Data underlying the findings described in this manuscript may be obtained in accordance with AstraZeneca’s data sharing policy described at: https://astrazenecagrouptrials.pharmacm.com/ST/Submission/Disclosure.
