# Supplementary material for: Anifrolumab in lupus nephritis: results from second-year extension of a randomised phase II trial
Source: Lupus Sci Med. 2023 Aug 22;10(2):e000910. doi: 10.1136/lupus-2023-000910 (PMC10445374; doi:10.1136/lupus-2023-000910)
Supplement: Supplementary data [file lupus-2023-000910supp001.pdf]

Supplementary to:

Anifrolumab in lupus nephritis: results from second year extension of a randomised phase 2 trial

David Jayne, MD, FRCP, Brad Rovin, MD, Eduardo Mysler, MD, Richard Furie, MD, Frédéric Houssiau, MD, PhD, Teodora Trasieva, MSc, Jacob Knagenhjelm, Erik Schwetje, BS, Weifeng Tang, MD, PhD, Raj Tummala, MD, MS, MBA, Catharina Lindholm, MD, PhD

SUPPLEMENTARY METHODS 3

Standard Therapy Protocols During Second-Year Extension Period 3

Oral glucocorticoids 3

Mycophenolate mofetil (MMF) 3

Study Endpoints 4

SUPPLEMENTARY TABLES 6

Table S1 Disease characteristics and oral glucocorticoid use at end of Year 1 of patients who continued in Year 2 6

Table S2 Non-serious adverse events during treatment above reporting threshold of 5% over the 2-year study period, by system organ class and preferred term 7

Table S3 Proportion of patients attaining individual components of the CRR and aCRR response at Week 104 9

SUPPLEMENTARY FIGURES 11

Figure S1 Flow chart of TULIP-LN trial design 11

Figure S2 Adjusted mean eGFR[a] over time 12

Figure S3 Proportion of patients with $CRR_{0.5}$ response over time 13

Figure S4 Probability of attaining a $CRR_{0.5}$ response sustained to Week 104[a] 14

Jayne D, *et al. Lupus Sci Med* 2024; 10:e000910. doi: 10.1136/lupus-2023-000910

Figure S5 Cumulative oral glucocorticoid dose up to Week 52 and up to Week 104, mITT population 15

Figure S6 Geometric mean 24-hour UPCR (mg/mg) by visit 16

Figure S7 Least squares mean change in baseline A) PGA and B) PtGA scores over time 17

Figure S8 Serological markers over time 19

Figure S9 Anifrolumab serum concentration profile over time 21

Figure S10 PD neutralisation of IFNGS over time 22

References 23

SUPPLEMENTARY METHODS

Standard Therapy Protocols During Second-Year Extension Period

Oral glucocorticoids

- If the oral glucocorticoid dosage was >7.5 mg/day at Week 52, tapering to ≤7.5 mg/day was required by Week 60. Failure to do so led to treatment discontinuation.
- Oral glucocorticoid dosage was required to be tapered to ≤5.0 mg/day at Week 80.
- Oral glucocorticoid tapering <5.0 mg/day was allowed at any time until Week 92.
- No change in oral glucocorticoid dosage was permitted from Week 92 to Week 104.
- One “burst and taper”, defined as either an oral glucocorticoid dose increase up to a maximum daily dose of 40 mg/day prednisone-equivalent dose for up to a total of 14 days that must be fully administered and tapered to ≤ the pre-burst starting dose by the end of the 15th day, or a maximum of 1 instance of intra-articular, tendon sheath, or bursal injections (for a total methylprednisolone ≤80 mg or equivalent), was permitted between Week 52 and Week 92.

Mycophenolate mofetil (MMF)

- The MMF dose was either ≤2 g/day or the Week-52 dose or below, whichever was lower.
- The MMF dose was not to be changed between Week 92 and Week 104.
- Reasons and consequences of changing the dose were as follows:
  - If the Week 52 dose was >2 g/day, the dose was to be tapered to ≤2 g/day by Week 60. Failure to comply led to withdrawal of investigational product.

Jayne D, *et al. Lupus Sci Med* 2024; 10:e000910. doi: 10.1136/lupus-2023-000910

- At the discretion of the Investigator, the dose of MMF could be decreased between Week 52 and Week 92.

At any time during the study, if MMF was discontinued and a different immunosuppressant was started, treatment was discontinued.

Study Endpoints

Prespecified efficacy endpoints included the relative difference in the mean change from baseline to Week 104 in 24-hour urine protein–creatinine ratio (UPCR) in the combined anifrolumab vs placebo groups (measured as a geometric mean ratio [GMR], with GMR<1 favouring anifrolumab); the proportion of patients at Week 104 attaining a complete renal response (CRR; defined as 24-hour UPCR ≤0.7 mg/mg, eGFR ≥60 mL/min/1.73 m^2 or no decrease ≥20% from baseline, no treatment discontinuation, and no restricted medication use beyond protocol-allowed threshold), PRR (defined previously), alternative CRR (aCRR, defined as a CRR with inactive urine sediment [<10 red blood cells per high-power field]), sustained oral glucocorticoid taper (≤5.0 mg/day prednisone equivalent from Weeks 80–104, among those receiving ≥20 mg/day at baseline) and CRR with sustained oral glucocorticoid taper. Prespecified endpoints also included the cumulative oral glucocorticoid dose; the mean change from baseline in non-renal SLE Disease Activity Index 2000 (SLEDAI-2K), [1] Physician's Global Assessment (PGA; measured on a visual analogue scale [VAS] ranging from 0–3 [2]), Patient's Global Assessment (PtGA),[3] and lupus serologies (anti-dsDNA antibodies, complement C3/C4); and the immunogenicity, pharmacokinetic (PK), and pharmacodynamic (PD) profiles of anifrolumab. PD neutralisation was measured as the median percentage change of baseline 21-gene type I IFNGS (21-IFNGS), as described previously. [4-7]

Post hoc efficacy analyses included cumulative proteinuria (the area under the curve in UPCR standardised by expected follow-up time), the proportion of patients with a $CRR_{0.5}$ (CRR requiring 24-UPCR ≤0.5 mg/mg) and the probability of obtaining a $CRR_{0.5}$ response sustained through Week 104.

Safety assessments included adverse events (AEs), laboratory assessments and vital signs. AEs of special interest (AESI) were non-opportunistic serious infections, opportunistic infections, herpes zoster (HZ), influenza, malignancy, tuberculosis, vasculitis, hypersensitivity, and major adverse cardiovascular events (MACEs).

Jayne D, *et al. Lupus Sci Med* 2024; 10:e000910. doi: 10.1136/lupus-2023-000910

SUPPLEMENTARY TABLES

Table S1 Disease characteristics and oral glucocorticoid use at end of Year 1 of patients who continued in Year 2

		Anifrolumab IR (n=29)	Anifrolumab BR (n=23)	Placebo (n=23)
**24-hour UPCR, mg/mg**	Mean (SD)	0.5 (0.5)	0.6 (0.5)	0.7 (0.7)
	>3.0, n (%)	0 (0.0)	0 (0.0)	0 (0.0)
**eGFR[a] mL/min/1.73 m^2**	Mean (SD)	100.5 (36.2)	96.7 (25.8)	76.6 (22.5)
	≥60, n (%)	26 (89.7)	21 (91.3)	18 (78.3)
**SLEDAI-2K[b] score**	Mean (SD)	4.6 (3.0)	5.0 (3.1)	5.9 (3.6)
**Non-renal SLEDAI-2K[b] score**	Mean (SD)	2.2 (2.0)	2.9 (1.6)	3.0 (1.4)
**Serology, n (%)**	Anti-dsDNA positive[c]	17 (58.6)	18 (78.3)	18 (78.3)
	Low C3[d]	8 (27.6)	9 (39.1)	11 (47.8)
	Low C4[d]	1 (3.4)	4 (17.4)	4 (17.4)
**Oral glucocorticoids[e], n (%)**	Yes, n (%)	26 (89.7)	19 (82.6)	21 (91.3)
	Dosage, mean (SD), mg/day	5.4 (2.8)	5.4 (3.5)	4.6 (2.8)

[a]eGFR is calculated using the MDRD formula; [b]The SLEDAI-2K is a 24-item weighted score of lupus activity that ranges from 0 to 105, with higher scores indicating greater disease activity; [c]Anti-dsDNA positive was defined as an anti-dsDNA level above the assay cutoff for positive; [d]Low complement level at baseline was defined as a complement level below lower limit of normal; [e]Prednisone or equivalent.

Anti-dsDNA, anti–double-stranded DNA; BR, basic regimen; C3, complement 3; C4, complement 4; eGFR, estimated glomerular filtration rate; IR, intensified regimen; MDRD, Modification of Diet in Renal Disease; SD, standard deviation; SLEDAI-2K, Systemic Lupus Erythematosus Disease Activity Index 2000; UPCR, urine protein–creatinine ratio.

Jayne D, *et al. Lupus Sci Med* 2024; 10:e000910. doi: 10.1136/lupus-2023-000910

Table S2 Non-serious adverse events during treatment above reporting threshold of 5% over the 2-year study period, by system organ class and preferred term

		Anifrolumab IR (n=51)		Anifrolumab BR (n=45)		Placebo (n=49)	
		n (%) of patients	Number of events	n (%) of patients	Number of events	n (%) of patients	Number of events
**Patients with any non-serious AE**		46 (90.2)		41 (91.1)		40 (81.6)	
**Patients with any non-serious AE at the threshold cutoff >5%**		39 (76.5)		31 (68.9)		33 (67.3)	
Infections and infestations		31 (60.8)		28 (62.2)		25 (51.0)	
	Urinary tract infection	6 (11.8)	8	10 (22.2)	11	5 (10.2)	7
	Nasopharyngitis	9 (17.6)	13	6 (13.3)	9	9 (18.4)	16
	Upper respiratory tract infection	7 (13.7)	9	8 (17.8)	17	8 (16.3)	10
	Bronchitis	7 (13.7)	7	4 (8.9)	4	6 (12.2)	6
	Herpes zoster	4 (7.8)	4	6 (13.3)	6	4 (8.2)	4
	Pharyngitis	4 (7.8)	5	3 (6.7)	5	2 (4.1)	2
	Oral herpes	3 (5.9)	3	3 (6.7)	4	2 (4.1)	6
	Herpes simplex	2 (3.9)	2	3 (6.7)	3	2 (4.1)	2
	Influenza	4 (7.8)	4	1 (2.2)	1	1 (2.0)	1
	Viral upper respiratory tract infection	3 (5.9)	3	1 (2.2)	2	0	0
Metabolism and nutrition disorders		3 (5.9)		1 (2.2)		0	
	Hyperglycaemia	3 (5.9)	3	1 (2.2)	1	0	0
Psychiatric disorders		0		1 (2.2)		3 (6.1)	
	Depression	0	0	1 (2.2)	1	3 (6.1)	3
Nervous system disorders		3 (5.9)		2 (4.4)		4 (8.2)	
	Headache	3 (5.9)	4	2 (4.4)	2	4 (8.2)	4
Vascular disorders		2 (3.9)		0		3 (6.1)	
	Hypertension	2 (3.9)	2	0	0	3 (6.1)	4
Respiratory, thoracic and mediastinal disorders		4 (7.8)		4 (8.9)		4 (8.2)	
	Cough	3 (5.9)	5	4 (8.9)	5	4 (8.2)	4
	Oropharyngeal pain	1 (2.0)	1	0	0	3 (6.1)	3
Gastrointestinal disorders		7 (13.7)		6 (13.3)		17 (34.7)	
	Diarrhoea	4 (7.8)	5	3 (6.7)	3	10 (20.4)	11
	Nausea	4 (7.8)	4	1 (2.2)	1	2 (4.1)	2
	Dyspepsia	0	0	2 (4.4)	2	4 (8.2)	4
	Vomiting	1 (2.0)	1	1 (2.2)	1	4 (8.2)	4
	Abdominal pain	0	0	0	0	4 (8.2)	6

Number (%) of patients who reported at least 1 non-serious AE for a preferred term at a frequency of >5% in any treatment group. Percentages are based upon all patients in the full analysis set.

An AE during treatment is defined as an AE with a date of onset ≥ day of first dose of treatment and ≤ date of last dose of treatment +28 days.

AEs were sorted by system organ class in international order, followed by descending frequency of preferred term in the combined anifrolumab groups.

Multiple occurrences of an AE in one patient in a preferred term were only counted once.

Adverse events are coded using MedDRA version 22.1.

AE, adverse event; BR, basic regimen; IR, intensified regimen; MedDRA, Medical Dictionary for Regulatory Activities.

Table S3 Proportion of patients attaining individual components of the CRR and aCRR response at Week 104

Patients With Response at Week 104, n (%)		Anifrolumab IR (n=44)	Anifrolumab BR (n=43)	Placebo (n=45)
CRR responder		12 (27.3)	8 (18.6)	8 (17.8)
aCRR responder		7 (15.9)	7 (16.3)	8 (17.8)
eGFR[a] ≥60 mL/min/1.73 m^2 or no decrease ≥20% from baseline, n (%)				
	Missing data[b]	24 (54.5)	28 (65.1)	29 (64.4)
	Nonresponder for eGFR[c]	0 (0)	0 (0)	1 (2.2)
	Responder for eGFR[c]	20 (45.5)	15 (34.9)	15 (33.3)
24-hour UPCR ≤0.7 mg/mg, n (%)				
	Missing data[b]	24 (54.5)	28 (65.1)	29 (64.4)
	Nonresponder for 24-hour UPCR[c]	6 (13.6)	2 (4.7)	6 (13.3)
	Responder for 24-hour UPCR[c]	14 (31.8)	13 (30.2)	10 (22.2)
Inactive urinary sediment (<10 RBC/hpf), n (%)				
	Missing data[b]	30 (68.2)	32 (74.4)	31 (68.9)
	Nonresponder for urinary sediment[c]	1 (2.3)	0 (0)	1 (2.2)
	Responder for urinary sediment[c]	13 (29.5)	11 (25.6)	13 (28.9)
Restricted medication use, n (%)				
	Discontinued treatment prior to visit	24 (54.5)	27 (62.8)	29 (64.4)
	Received restricted medications[d]	3 (6.8)	6 (14.0)	4 (8.9)
	Did not receive restricted medications[d]	17 (38.6)	10 (23.3)	12 (26.7)
Discontinued treatment				
	Discontinued	24 (54.5)	27 (62.8)	29 (64.4)
	Did not discontinue	20 (45.5)	16 (37.2)	16 (35.6)

Patients from Italy and France were not included in the analysis and are not included in the percentage calculations. A CRR required 24-hour UPCR ≤0.7 mg/mg, eGFR ≥60 mL/min/1.73 m^2 or no decrease ≥20% from baseline, no treatment discontinuation and no use of restricted medications. An aCRR required all the components of the CRR definition and inactive urinary sediment (<10 RBC/hpf).

[a]eGFR was calculated with the MDRD formula. [b]Missing data, irrespective of treatment adherence; therefore, not possible to evaluate response. [c]Including patients who discontinued treatment. [d]Not including patients who discontinued treatment.

aCRR, alternative CRR, complete renal response with inactive urinary sediment; BR, basic regimen; CRR, complete renal response; eGFR, estimated glomerular filtration rate; hpf, high-power field; IR, intensified regimen; MDRD, Modification of Diet in Renal Disease; RBC, red blood cell; UPCR, urine protein–creatinine ratio.

SUPPLEMENTARY FIGURES

Figure S1 Flow chart of TULIP-LN trial design

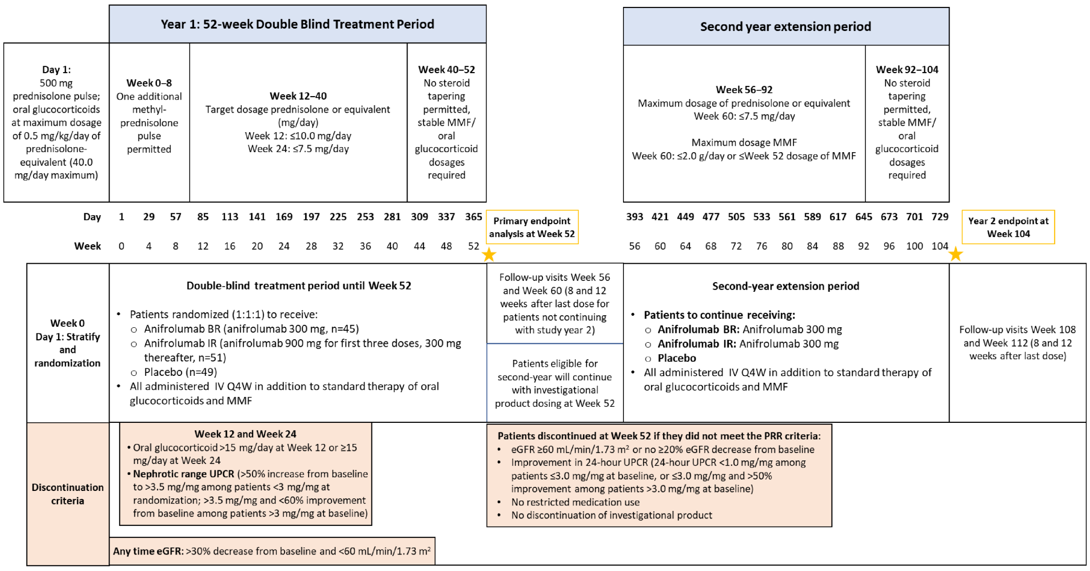

BR, basic regimen, eGFR, estimated glomerular filtration rate; IR, intensified regimen; IV, intravenous; LN, lupus nephritis; MMF, mycophenolate mofetil; PRR, partial renal response; Q4W, every 4 weeks; UPCR, urine protein–creatinine ratio.

Reproduced from Jayne D, et al. *Ann Rheum Dis.* 2022. doi:10.1136/annrheumdis-2021-221478 with permission from BMJ Publishing Group Ltd.

Figure S2 Adjusted mean eGFR[a] over time

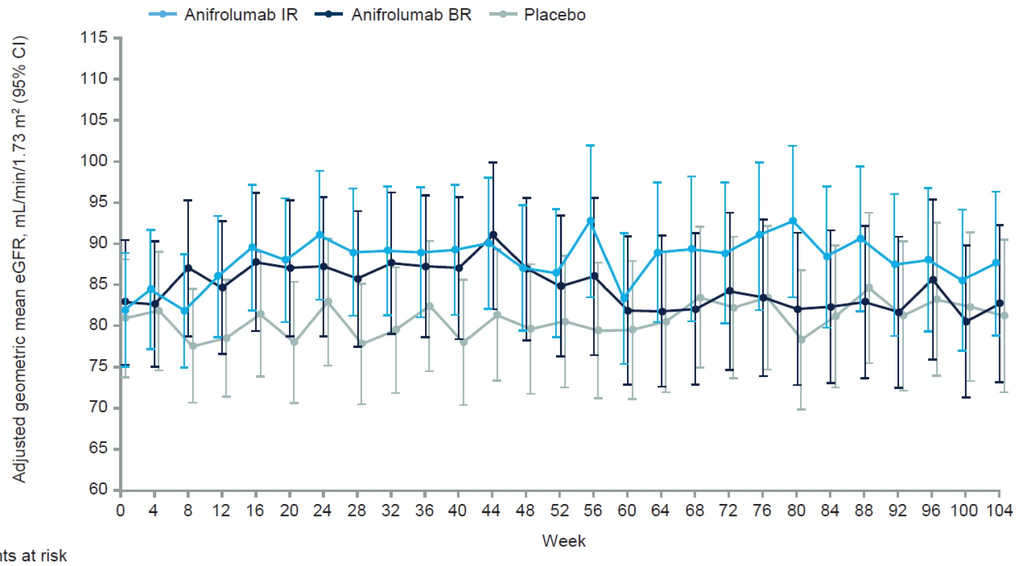

Patients at risk	0	4	8	12	16	20	24	28	32	36	40	44	48	52	56	60	64	68	72	76	80	84	88	92	96	100	104
Anifrolumab IR	51	49	50	49	49	48	48	42	42	39	41	39	40	38	26	29	29	25	27	26	27	26	26	24	24	24	23
Anifrolumab BR	45	43	41	41	41	40	35	37	34	33	31	29	31	29	20	20	19	19	18	18	19	19	19	19	18	17	16
Placebo	49	46	45	41	37	35	35	32	31	30	28	27	27	26	23	22	21	22	22	22	20	20	19	17	17	18	16

Data points are adjusted mean eGFR at the respective week, controlled for baseline and stratification factors, with 95% confidence intervals.

[a]eGFR was estimated based on creatinine ratio.

BR, basic regimen; CI, confidence interval; eGFR, estimated glomerular filtration rate; IR, intensified regimen.

Figure S3 Proportion of patients with $CRR_{0.5}$ response over time

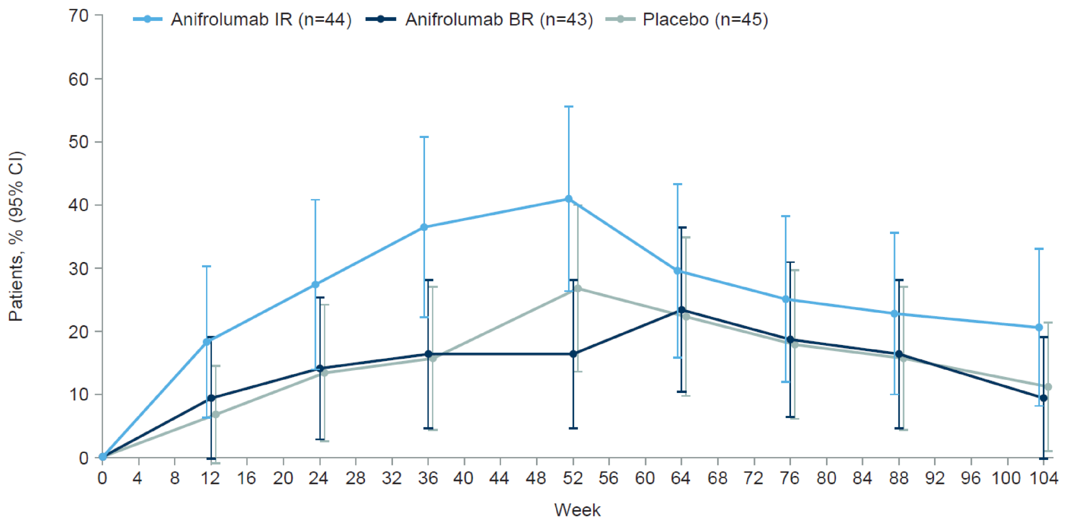

A $CRR_{0.5}$ required 24-hour UPCR ≤0.5 mg/mg, eGFR ≥60 mL/min/1.73 m^2 or no decrease ≥20% from baseline, no treatment discontinuation and no use of restricted medications. $CRR_{0.5}$ was analysed post hoc.

The response rates were calculated with a weighted Cochran–Mantel–Haenszel method using the full analysis set but excluding patients from France and Italy. Percentages are based on the number of patients in the analysis, so the denominator remains the same each week (anifrolumab IR, n=44; anifrolumab BR, n=43; placebo, n=45).

BR, basic regimen; CI, confidence interval; $CRR_{0.5}$, complete renal response with UPCR ≤0.5 mg/mg; eGFR, estimate glomerular filtration rate; IR, intensified regimen; UPCR, urine protein–creatinine ratio.

Jayne D, *et al. Lupus Sci Med* 2024; 10:e000910. doi: 10.1136/lupus-2023-000910

Figure S4 Probability of attaining a $CRR_{0.5}$ response sustained to Week 104[a]

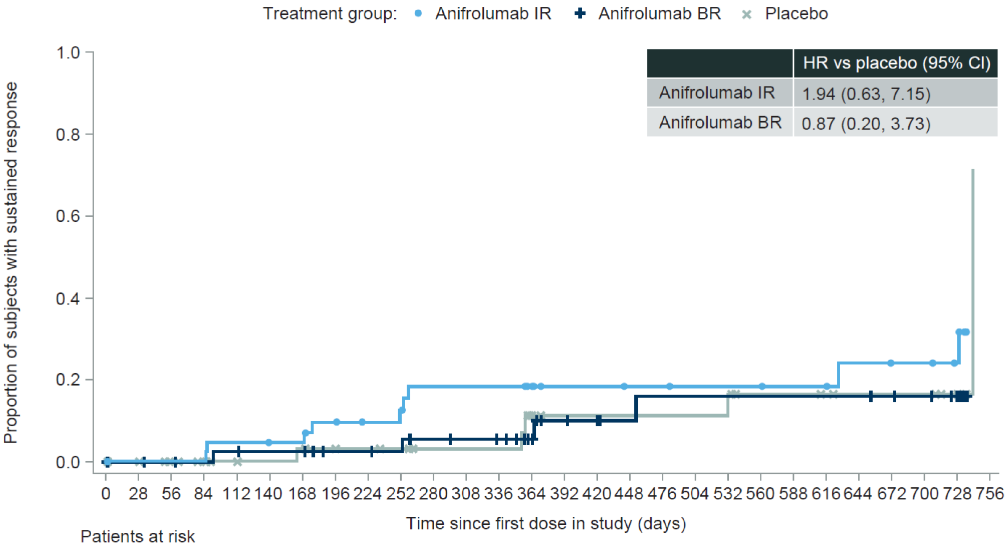

Patients at risk

Study Days	0	28	56	84	112	140	168	196	224	252	280	308	336	364	392	420	448	476	504	532	560	588	616	644	672	700	728	756
Anifrolumab IR	44	43	43	43	41	40	40	34	32	31	28	28	28	25	18	18	17	17	16	16	16	15	15	13	12	12	10	0
Anifrolumab BR	43	42	41	40	39	38	38	33	33	32	29	28	27	22	18	17	15	14	14	14	14	14	14	14	13	12	10	0
Placebo	45	42	39	37	33	32	31	28	27	26	24	24	24	20	17	17	17	17	17	17	14	14	13	12	12	12	9	0

[a]$CRR_{0.5}$ required 24-hour UPCR ≤0.5 mg/mg, eGFR ≥60 mL/min/1.73 m^2 or no decrease ≥20% from baseline, no treatment discontinuation and no use of restricted medications. Probability of obtaining a sustained $CRR_{0.5}$ was analysed post hoc using a Cox regression model controlling for stratification factors, using the overall mITT population but excluding patients from France and Italy.

BR, basic regimen; CI, confidence interval; $CRR_{0.5}$, complete renal response with UPCR ≤0.5 mg/mg; eGFR, estimate glomerular filtration rate; HR, hazard ratio; IR, intensified regimen; mITT, modified intention-to-treat; UPCR, urine protein–creatinine ratio.

Figure S5 Cumulative oral glucocorticoid dose up to Week 52 and up to Week 104, mITT population

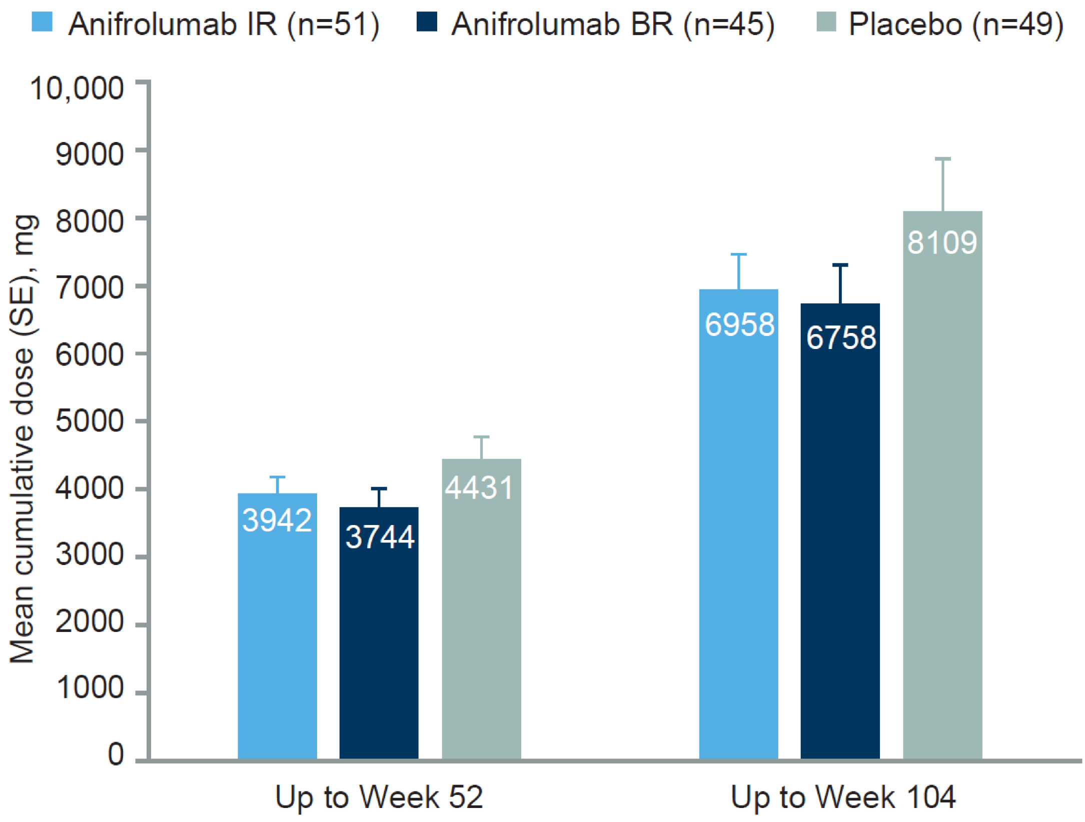

Cumulative oral glucocorticoid dose was calculated as the standardised area under the curve of oral glucocorticoid dose (mg). All data up to and including the date of treatment discontinuation were included in the analysis.

BR, basic regimen; IR, intensified regimen; mITT, modified intention-to-treat; SE, standard error.

Figure S6 Geometric mean 24-hour UPCR (mg/mg) by visit

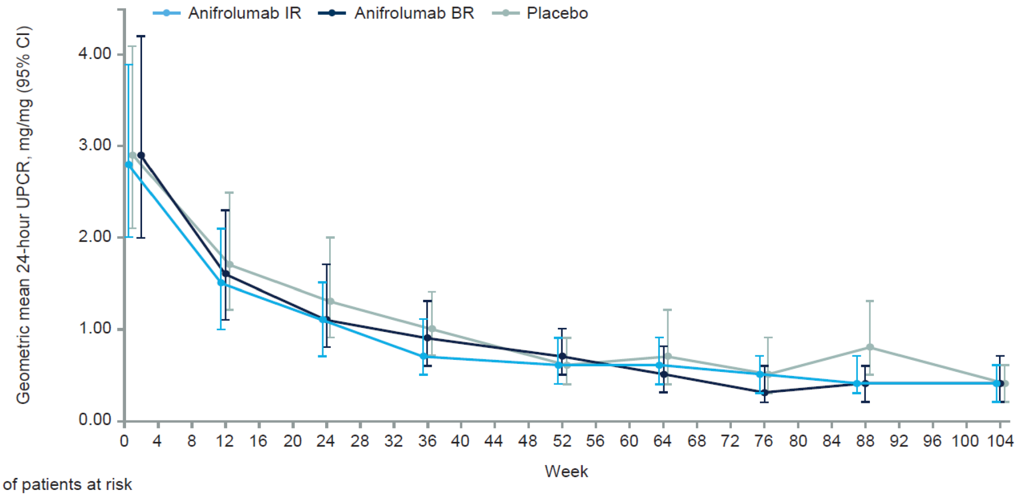

Number of patients at risk									
Anifrolumab IR	51	43	45	32	37	19	20	22	18
Anifrolumab BR	45	35	35	30	29	14	15	15	13
Placebo	49	36	32	28	25	14	18	15	13

The model includes fixed effects for treatment group, visit, stratification factors (IFNGS high vs low at screening and 24-hour UPCR 7–14 days prior to screening ≤3 or >3 mg/mg), log-transformed 24-hour UPCR at baseline and treatment-by-visit interaction.

BR, basic regimen; CI, confidence interval; IFNGS, interferon gene signature; IR, intensified regimen; UPCR, urine protein–creatinine ratio.

Figure S7 Least squares mean change in baseline A) PGA and B) PtGA scores over time

A. Mean change in PGA score

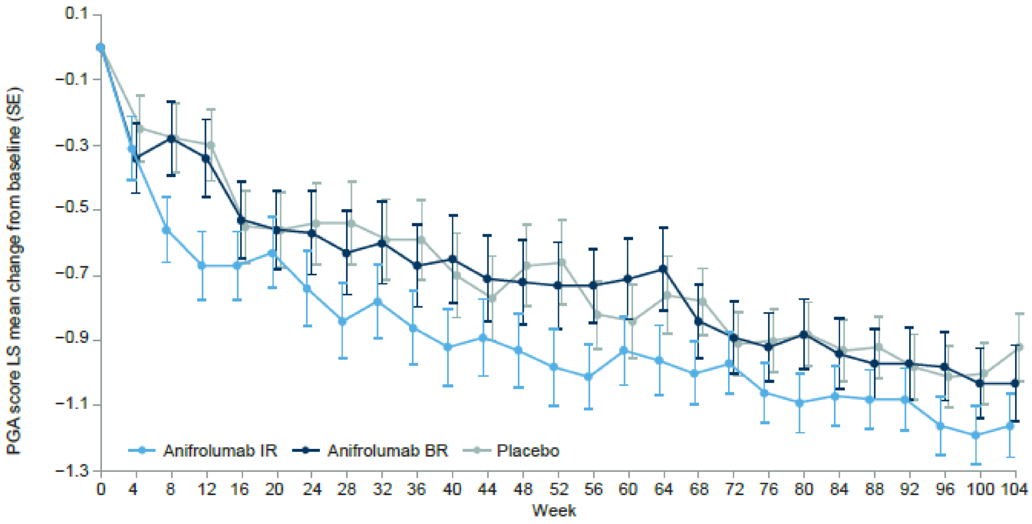

Patients at risk																										
Anifrolumab IR	46	50	48	48	48	48	43	42	38	40	39	39	35	29	29	29	26	27	25	27	26	26	24	23	23	24
Anifrolumab BR	42	41	40	39	38	37	36	35	33	30	26	26	26	20	18	18	18	17	18	19	18	18	17	18	16	14
Placebo	44	42	39	35	33	34	31	31	30	28	25	26	26	22	21	20	22	22	21	20	20	20	17	16	18	17

B. Mean change in PtGA score

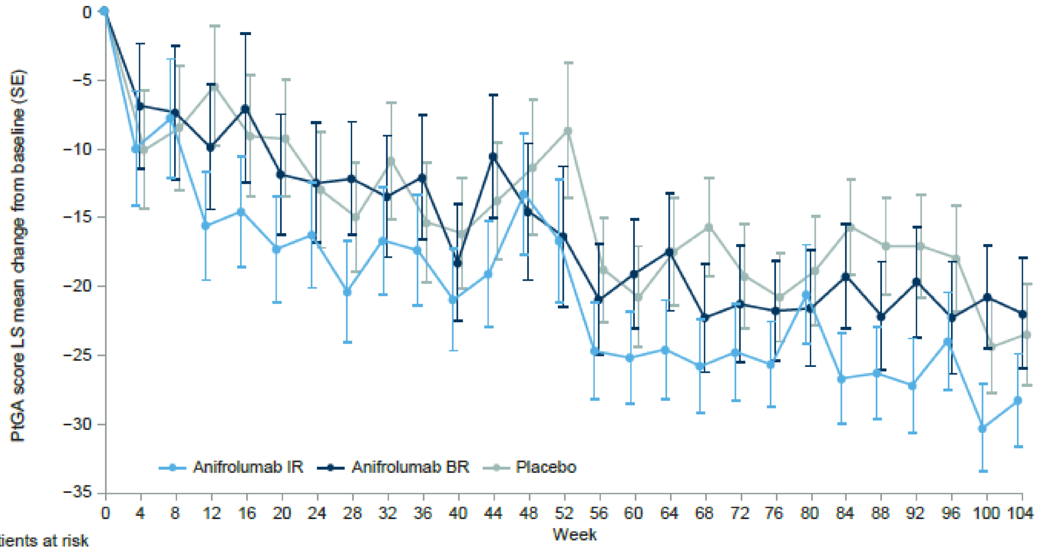

Patients at risk																										
Anifrolumab IR	45	48	49	48	46	47	43	41	38	40	40	37	34	29	29	29	26	27	25	27	26	26	23	22	22	24
Anifrolumab BR	41	38	38	39	37	36	36	33	33	29	29	30	25	20	18	17	19	17	17	19	19	17	16	17	17	16
Placebo	43	43	36	35	33	33	30	30	30	28	27	26	25	21	19	20	21	21	21	20	20	20	17	16	18	16

PGA and PtGA least squares mean change from baseline were analysed using a mixed model for repeated measures, including fixed effects for treatment group, visit, stratification factors, baseline value and treatment-by-visit interaction. All data up to and including the date of treatment discontinuation were included in the analysis.

BR, basic regimen; IR, intensified regimen; LS, least squares, PGA, Physician's Global Assessment, PtGA, Patient's Global Assessment; SE, standard error.

Jayne D, *et al. Lupus Sci Med* 2024; 10:e000910. doi: 10.1136/lupus-2023-000910

Figure S8 Serological markers over time

A. Median change from baseline anti-dsDNA (U/mL)

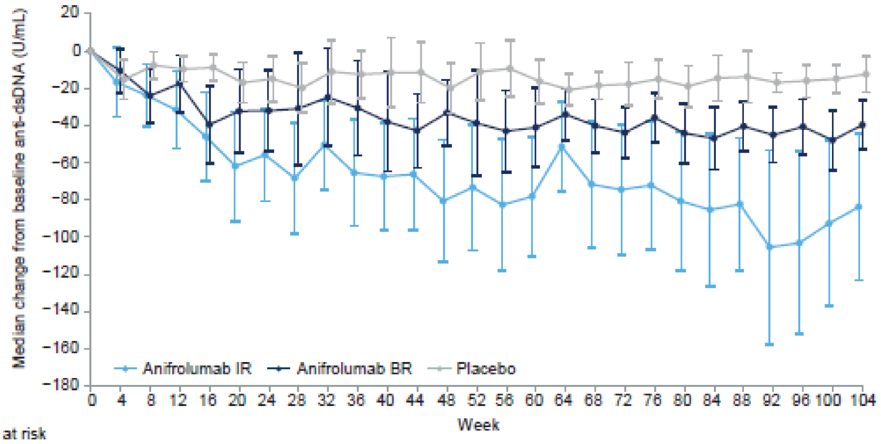

Patients at risk																											
Anifrolumab IR	39	34	38	36	36	35	35	36	37	37	39	38	37	33	26	33	23	19	21	22	22	21	20	18	18	17	17
Anifrolumab BR	37	34	35	36	35	34	33	34	34	34	31	29	31	29	21	23	16	16	15	14	14	14	14	14	13	14	11
Placebo	39	32	36	34	37	36	33	35	32	33	34	32	31	30	23	24	15	18	18	17	17	16	15	12	12	13	11

B. Median change from baseline C3 (g/L)

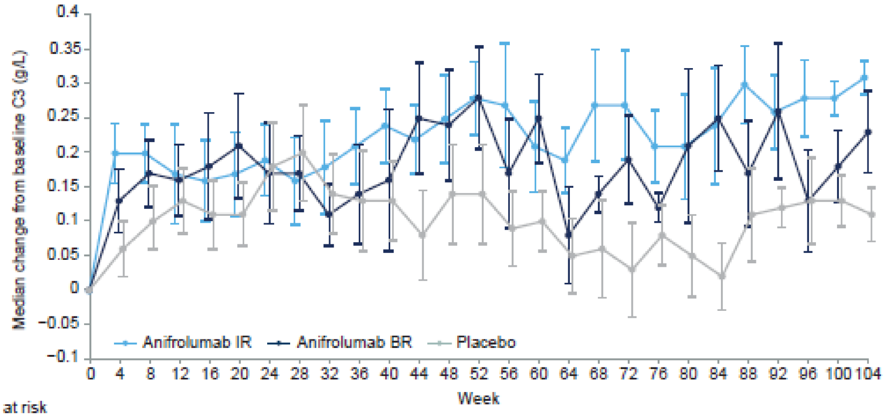

Patients at risk																											
Anifrolumab IR	27	26	27	27	26	25	26	25	25	26	27	26	25	24	20	24	19	12	15	15	15	15	14	14	14	13	13
Anifrolumab BR	30	30	29	28	28	29	23	28	25	27	25	23	25	23	16	18	15	13	12	11	11	10	11	10	11	11	9
Placebo	42	40	39	37	38	38	36	36	35	34	36	32	32	33	24	23	17	17	18	17	17	16	15	11	12	13	11

C. Median change from baseline C4 (g/L)

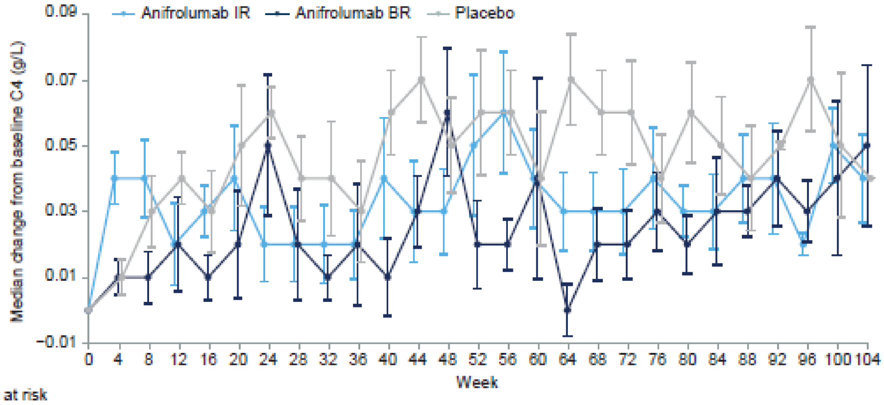

Patients at risk																											
Anifrolumab IR	14	13	13	14	13	13	13	13	14	13	14	13	13	12	10	12	10	6	8	8	8	8	8	8	8	8	8
Anifrolumab BR	10	10	10	10	9	10	7	10	8	9	10	9	9	8	7	7	5	5	5	4	4	4	4	3	4	4	4
Placebo	20	20	18	16	18	17	16	16	16	15	16	14	13	14	8	9	4	4	5	4	4	4	4	3	3	4	1

Abnormal (low) complement at baseline is defined as complement level below the lower limit of normal and/or abnormal (positive) anti-dsDNA at baseline.

Points are medians and error bars are median absolute deviations.

anti-dsDNA, anti–double-stranded DNA; BR, basic regimen; C3, complement 3; C4, complement 4; IR, intensified regimen.

Jayne D, *et al. Lupus Sci Med* 2024; 10:e000910. doi: 10.1136/lupus-2023-000910

Figure S9 Anifrolumab serum concentration profile over time

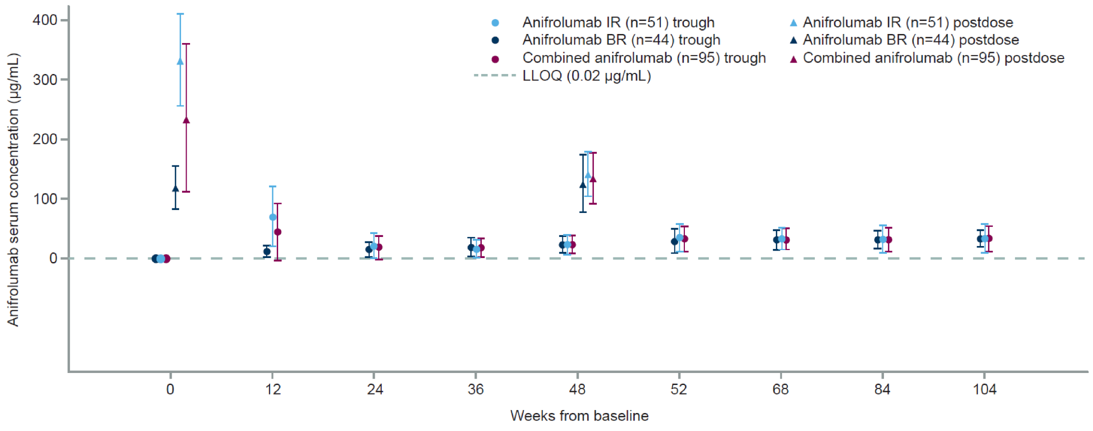

Pharmacokinetics were analysed in the 95 patients who received ≥1 dose of anifrolumab. Data points are average observed steady-state serum trough (pre-dose) concentrations. Maximum post-dose concentrations after the first dose are also displayed.

BR, basic regimen; IR, intensified regimen; LLOQ, lower limit of quantification; N, number of subjects in treatment group.

Figure S10 PD neutralisation of IFNGS over time

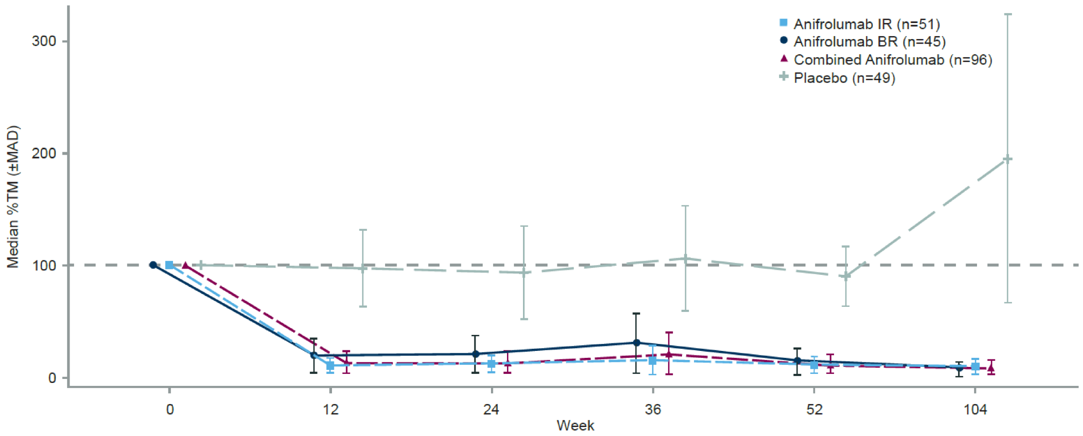

Patients at risk						
Anifrolumab IR	51	45	43	40	35	21
Anifrolumab BR	45	39	35	38	30	13
Combined Anifrolumab	96	84	78	78	65	34
Placebo	49	37	35	34	31	10

Pharmacodynamic analysis was only carried out on the 137 patients who were type I IFNGS-high at screening. Points represent the median percent of baseline 21-IFNGS expression ± MAD.

BR, basic regimen; IFNGS, interferon gene signature; IR, intensified regimen; MAD, median absolute deviation; n, number of non-missing values; PD, pharmacodynamic; %TM, percent target modulation.

References

1 Gladman DD, Ibañez D, Urowitz MB. Systemic lupus erythematosus disease activity index 2000. *J Rheumatol* 2002;29:288–91. https://doi.org/https://www.ncbi.nlm.nih.gov/pubmed/11838846.

2 Petri M, Genovese M, Engle E, et al. Definition, incidence, and clinical description of flare in systemic lupus erythematosus. A prospective cohort study. *Arthritis Rheum* 1991;34:937–44. https://doi.org/10.1002/art.1780340802.

3 Liang MH, Socher SA, Larson MG, et al. Reliability and validity of six systems for the clinical assessment of disease activity in systemic lupus erythematosus. *Arthritis Rheum* 1989;32:1107–18. https://doi.org/10.1002/anr.1780320909.

4 Yao Y, Higgs BW, Morehouse C, et al. Development of potential pharmacodynamic and diagnostic markers for anti-IFN-α monoclonal antibody trials in systemic lupus erythematosus. *Hum Genomics Proteomics* 2009;2009:374312. https://doi.org/10.4061/2009/374312.

5 Yao Y, Higgs BW, Richman L, et al. Use of type I interferon-inducible mRNAs as pharmacodynamic markers and potential diagnostic markers in trials with sifalimumab, an anti-IFNα antibody, in systemic lupus erythematosus. *Arthritis Res Ther* 2010;12:S6. https://doi.org/10.1186/ar2887.

6 Furie R, Khamashta M, Merrill JT, et al. Anifrolumab, an anti-interferon-α receptor monoclonal antibody, in moderate-to-severe systemic lupus erythematosus. *Arthritis Rheumatol* 2017;69:376–86. https://doi.org/10.1002/art.39962.

7 Jayne D, Rovin B, Mysler EF, et al. Phase II randomised trial of type I interferon inhibitor anifrolumab in patients with active lupus nephritis. *Ann Rheum Dis* 2022;81:496–506. https://doi.org/10.1136/annrheumdis-2021-221478.
